# High levels of tumor cell-intrinsic STING signaling are associated with increased infiltration of CD8^+^ T cells in dMMR/MSI-H gastric cancer

**DOI:** 10.1038/s41598-024-71974-3

**Published:** 2024-09-06

**Authors:** Ryo Kanoda, Shotaro Nakajima, Satoshi Fukai, Motonobu Saito, Katsuharu Saito, Hiroya Suzuki, Tomohiro Kikuchi, Azuma Nirei, Hirokazu Okayama, Kosaku Mimura, Hiroyuki Hanayama, Wataru Sakamoto, Tomoyuki Momma, Zenichiro Saze, Koji Kono

**Affiliations:** 1 Department of Gastrointestinal Tract Surgery, Fukushima Medical University School of Medicine, Fukushima, Japan; 2 Department of Multidisciplinary Treatment of Cancer and Regional Medical Support, Fukushima Medical University School of Medicine, 1 Hikariga-Oka, Fukushima City, Fukushima 960-1295 Japan; 3 Department of Blood Transfusion and Transplantation Immunology, Fukushima Medical University School of Medicine, Fukushima, Japan

**Keywords:** Gastric cancer, Mismatch repair deficient, Cyclic GMP–AMP synthase–stimulator of interferon genes, CD8^+^ tumor-infiltrating lymphocytes, Tumor microenvironment, Gastric cancer, Cancer microenvironment, Tumour immunology

## Abstract

Mismatch repair deficient (dMMR)/microsatellite instability-high (MSI-H) gastric cancer (GC) exhibits an immune-active tumor microenvironment (TME) compared to MMR proficient (pMMR)/microsatellite stable/Epstein-Barr virus-negative [EBV (−)] GC. The tumor cell-intrinsic cyclic GMP–AMP synthase (cGAS)–stimulator of interferon genes (STING) pathway has been considered a key regulator of immune cell activation in the TME. However, its significance in regulating the immune-active TME in dMMR/MSI-H GC remains unclear. Here, we demonstrated that tumor cell-intrinsic cGAS–STING was highly expressed in dMMR GC compared to pMMR/EBV (−) GC. The expression of tumor cell-intrinsic STING was significantly and positively associated with the number of CD8^+^ tumor-infiltrating lymphocytes in GC. Analysis of TCGA datasets revealed that the expression of interferon-stimulated genes and STING downstream T-cell attracting chemokines was significantly higher in MSI-H GC compared to other subtypes of GC with EBV (−). These results suggest that tumor cell-intrinsic STING signaling plays a key role in activating immune cells in the dMMR/MSI-H GC TME and might serve as a novel biomarker predicting the efficacy of immunotherapy for GC treatment.

## Introduction

Gastric cancer (GC) ranks as the third leading cause of cancer-related deaths worldwide^1,2^. Despite advancements in multidisciplinary treatments that have improved clinical outcomes, prognosis for patients with advanced GC remains poor, highlighting the need for further therapeutic strategies. The Cancer Genome Atlas (TCGA) proposed a molecular classification that categorizes GC into four distinct subtypes: microsatellite unstable/instability-high (MSI-H), Epstein-Barr virus-positive [EBV (+)], genomically stable (GS), and chromosomal instability (CIN) tumors^3^. MSI-H and EBV (+) GCs are recognized as tumors with an immune-active tumor microenvironment (TME)^4^. Loss of DNA mismatch repair (MMR; MMR deficient [dMMR]) leads to the MSI-H phenotype, accounting for 20–25% and 8–19% of all GC cases in Western and Asian countries, respectively^5^. In the sporadic setting, more than 50% of dMMR/MSI-H GC cases arise from hypermethylation of the promoter region of *MutL homolog 1* (*MLH1*), the most critical MMR gene, while mutations in MMR genes, including *MLH1* and *mutS homolog 2*, are obsereved in approximately 15% of dMMR/MSI-H GC cases^6^. dMMR/MSI-H GC is characterized by high tumor mutational burden, neoantigen load, dense infiltration of immune cells such as CD8^+^ T cells, and higher expression of programmed cell death ligand 1 (PD-L1) compared to MMR proficient (pMMR)/microsatellite stable (MSS)/EBV (−) GC. Indeed, dMMR/MSI-H GC likely exhibits a better prognosis and a favorable response to immune checkpoint inhibitors (ICIs), such as anti-PD-1/PD-L1 agents, compared to pMMR/MSS/EBV (−) GC^7–10^. However, the regulatory mechanisms of the immune-active TME in dMMR/MSI-H GC remain incompletely understood.

The cyclic GMP–AMP synthase (cGAS)–stimulator of interferon genes (STING) pathway has been recognized as a key regulator of the tumor-immune microenvironment in various cancer types, including GC^11,12^. In response to abnormally exposed double-stranded DNA in cytoplasm of cancer cells, activated cGAS synthesizes cGAMP, a second messenger, which binds to and activates STING signaling. This activation results in the induction of type I interferon (IFN) response and the upregulation of pro-inflammatory cytokines and immunostimulatory chemokines, including C–X–C motif ligand (CXCL) 9/10/11 and C–C motif chemokine ligand 5 (CCL5)^13^. Previous studies have revealed that the downregulation of tumor cell-intrinsic STING signaling is significantly associated with decreased intra-tumoral infiltration and activation of antigen-presenting cells and CD8^+^ T cells in various cancer types^14–16^. Song et al*.* reported that the tumor cell-intrinsic expression of STING was significantly decreased in advanced GC, and their in vitro experiments demonstrated that the knockdown of STING promoted GC cell survival^17^. Therefore, the cGAS–STING pathway could be considered a crucial signaling pathway activating immune cells in the GC TME.

In this study, to investigate the role of the tumor cell-intrinsic cGAS–STING pathway in the immune-active TME of dMMR GC, we assessed the tumor cell-intrinsic expression of cGAS–STING between pMMR/EBV (−) and dMMR GCs through immunohistochemistry (IHC) analysis of our own GC cohort. Additionally, we evaluated the association between the tumor cell-intrinsic cGAS–STING expression and CD8^+^ T cell-infiltration in both pMMR/EBV (−) and dMMR GCs.

## Results

### High expression of the tumor cell-intrinsic cGAS–STING in dMMR GC

**Fig. 1:**
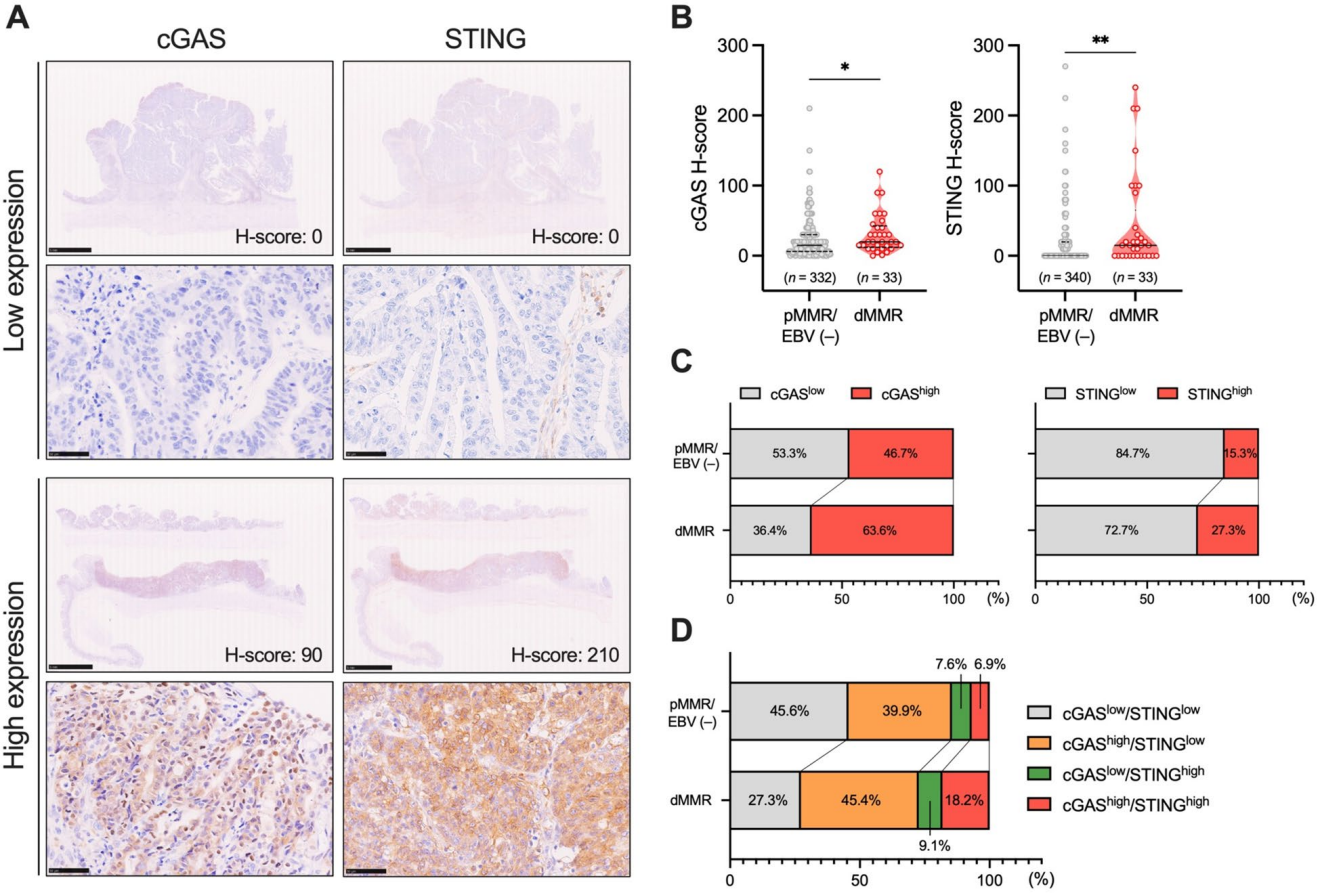
Relationship between the tumor cell-intrinsic expression of cGAS–STING and MMR status in GC. (**A**) Representative IHC images showing cGAS–STING expression in GCs with high and low levels. Scale bars: 5 mm for low magnifications and 50 μm for high magnifications. (**B**) Comparison of H-scores for cGAS–STING between pMMR/EBV (−) and dMMR GCs. Solid lines represent medians, while dotted lines represent quantiles. (**C**) Percentages of cases with low or high tumor cell-intrinsic expression of cGAS (cGAS^low^ or cGAS^high^) and STING (STING^low^ or STING^high^) in pMMR/EBV (−) and dMMR GCs. (**D**) Percentages of cases with cGAS^low^/STING^low^, cGAS^high^/STING^low^, cGAS^low^/STING^high^, and cGAS^high^/STING^high^ in pMMR/EBV (−) and dMMR GCs. Statistical significance was determined by the Mann–Whitney U test (**B**). *p < 0.05, **p < 0.01.

We initially examined the differences in clinicopathological features between pMMR/EBV (−) and dMMR GCs. As demonstrated in Table 1, consistent with previous reports^18^, dMMR GC was significantly associated with older age (*p* < 0.0001), distal tumor location (*p* = 0.0005), and higher PD-L1 expression (*p* = 0.0025) compared to pMMR/EBV (−) GC. We also assessed the association of tumor cell-intrinsic expression of cGAS–STING with MMR status in GC. Figure 1A displays representative IHC images for the expression of cGAS and STING in GCs with high and low expression. We observed that H-scores of cGAS and STING were significantly higher in dMMR GC compared to those in pMMR/EBV (−) GC (Fig. 1B). The frequencies of cGAS^high^, STING^high^, and cGAS^high^/STING^high^ GCs were higher in dMMR GC compared to pMMR/EBV (−) GC (Fig. 1C, D), suggesting that the tumor cell-intrinsic expression of cGAS–STING is up-regulated in dMMR GC.

**Table 1:** Clinicopathological characteristics of patients with gastric cancer.

	Total	pMMR/EBV (−)	dMMR	EBV (+)	*p*-value^§^	*p*-value^§§^	*p*-value^§§§^
	*n* = 401	*n* = 341 (85.1%)	*n* = 33 (8.2%)	*n* = 27 (6.7%)			
Age					< 0.0001	0.6158	0.0009
Mean ± SD	76.0 ± 11.0	67.1 ± 11.1	75.8 ± 7.9	66.5 ± 9.8			
Gender					0.4254	0.8281	0.4186
Male	283 (70.6%)	242 (71.0%)	21 (63.6%)	20 (74.1%)			
Female	118 (29.4%)	99 (29.0%)	12 (36.4%)	7 (25.9%)			
Location					0.0005	0.0054	< 0.0001
Upper	129 (32.2%)	110 (32.3%)	3 (9.1%)	16 (59.3%)			
Middle	131 (32.7%)	116 (34.0%)	7 (21.2%)	8 (29.6%)			
Low	124 (30.9%)	103 (30.2%)	20 (60.6%)	1 (3.7%)			
Remnant GC	17 (4.2%)	12 (3.5%)	3 (9.1%)	2 (7.4%)			
Histological type					0.2021	0.4217	0.1215
Differentiated	203 (50.6%)	171 (50.1%)	21 (63.6%)	11 (40.7%)			
Undifferentiated	191 (47.7%)	164 (48.1%)	12 (36.4%)	15 (55.6%)			
Unclear	7 (1.7%)	6 (1.8%)	0 (0%)	1 (3.7%)			
Tumor invasion					0.6901	0.0646	0.5405
T1	204 (50.9%)	182 (53.4%)	14 (42.4%)	8 (29.6%)			
T2	51 (12.7%)	43 (12.6%)	4 (12.1%)	4 (14.9%)			
T3	44 (11.0%)	36 (10.5%)	5 (15.2%)	3 (11.1%)			
T4	101 (25.2%)	80 (23.5%)	9 (27.3%)	12 (44.4%)			
Unclear	1 (0.2%)	0 (0%)	1 (3.0%)	0 (0%)			
Lymph node metastasis				0.8530	0.8403	0.7932	
Present	155 (38.7%)	132 (38.7%)	12 (36.4%)	11 (40.7%)			
Absent	245 (61.1%)	208 (61.0%)	21 (63.6%)	16 (59.3%)			
Unclear	1 (0.2%)	1 (0.3%)	0 (0%)	0 (0%)			
Distant metastasis					> 0.9999	0.4870	0.6494
Present	33 (8.2%)	28 (8.2%)	2 (6.1%)	3 (11.1%)			
Absent	368 (91.8%)	313 (91.8%)	31 (93.9%)	24 (88.9%)			
pTNM Stage					0.8369	0.3610	0.8585
I	219 (54.6%)	192 (56.3%)	16 (48.5%)	11 (40.7%)			
II	79 (19.7%)	65 (19.1%)	8 (24.3%)	6 (22.2%)			
III	70 (17.5%)	58 (17.0%)	6 (18.1%)	6 (22.2%)			
IV	33 (8.2%)	26 (7.6%)	3 (9.1%)	4 (14.9%)			
HER2					0.2285	> 0.9999	0.5834
Positive	39 (9.7%)	36 (10.6%)	1 (3.0%)	2 (7.4%)			
Negative	362 (90.3%)	305 (89.4%)	32 (97.0%)	25 (92.6%)			
PD-L1 (CPS ≧ 5)					0.0025	0.0013	0.0334
Positive	138 (34.4%)	95 (27.9%)	18 (54.5%)	25 (92.6%)			
Negative	263 (65.6%)	246 (72.1%)	15 (45.5%)	2 (7.4%)			
MMR status					–	–	–
Deficient	33 (8.2%)	0 (0%)	33 (3.0%)	0 (0%)			
Proficient	368 (91.8%)	341 (100%)	0 (0%)	27 (100%)			
EBV status					–	–	–
Positive	27 (6.7%)	0 (0%)	0 (0%)	27 (100%)			
Negative	374 (93.3%)	341 (100%)	33 (100%)	0 (92.6%)			

Data are presented as number (%) unless otherwise indicated.

CPS, combined positive score; EBV, Epstein-Barr virus; GC, gastric cancer; HER2, human epidermal receptor 2; dMMR, mismatch repair deficient; pMMR, mismatch repair proficient; PD-L1, programmed cell death ligand 1; pTNM, pathological tumor-node-metastasis; SD, standard deviation.

*Statistically significant, *p* < 0.05.

^§^pMMR/EBV (−) versus dMMR.

^§§^pMMR/EBV (−) versus EBV (+).

^§§§^dMMR versus EBV (+).

### Involvement of the tumor cell-intrinsic STING expression in the high infiltration of CD8^+^ T cells in dMMR GC

**Fig. 2:**
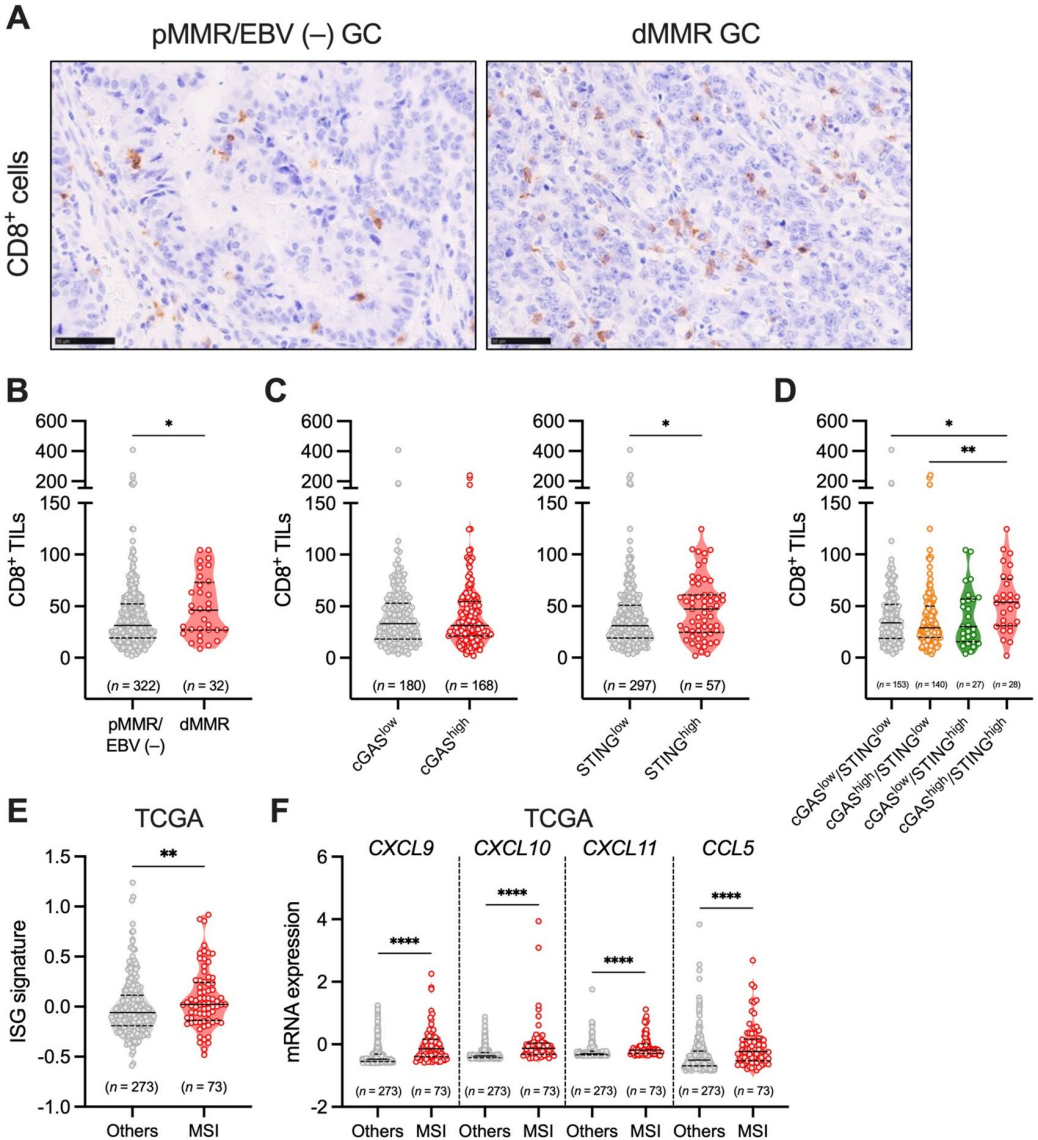
Relationship between the number of CD8^+^ TILs, MMR status, and the tumor cell-intrinsic expression of cGAS–STING in GC. (**A**) Representative IHC images of CD8 in pMMR/EBV (−) and dMMR GCs. Scale bars: 50 μm. (**B**) Comparison of the number of CD8^+^ TILs between pMMR/EBV (−) and dMMR GCs. Solid lines represent medians, while dotted lines represent quantiles. (**C**) Comparisons of the number of CD8^+^ TILs between cGAS^low^ and cGAS^high^ GCs or STING^low^ and STING^high^ GCs. (**D**) Comparison of the number of CD8^+^ TILs among cGAS^low^/STING^low^, cGAS^high^/STING^low^, cGAS^low^/STING^high^, and cGAS^high^/STING^high^ GCs. (**E**) Comparison of the expression of ISG signature between MSI-H GC and other subtypes of GC with EBV (−) (TCGA cohort). (**F**) Comparison of the expression of *CXCL9/10/11* and *CCL5* between MSI-H GC and other subtypes of GC with EBV (−) (TCGA cohort). Statistical significance was determined by the Kruskal–Wallis test with Dunn’s multiple comparisons test (**D**), and the Mann–Whitney U test (**B**, **C**, **E**, **F**). *p < 0.05, **p < 0.01, ****p < 0.0001.

We then assessed the number of CD8^+^ TILs through IHC analysis of our GC cohort. We revealed a significantly higher number of CD8^+^ TILs in dMMR GC compared to pMMR/EBV (−) GC (Fig. 2A, B). Furthermore, we observed a significant positive correlation between tumor cell-intrinsic expression of STING, but not cGAS, and the number of CD8^+^ TILs in GCs (Fig. 2C). When comparing the number of CD8^+^ TILs among patients with cGAS^low^/STING^low^, cGAS^high^/STING^low^, cGAS^low^/STING^high^, and cGAS^high^/STING^high^ GCs, those with cGAS^high^/STING^high^ GC exhibited the highest number of CD8^+^ TILs (Fig. 2D).

Analysis of TCGA GC dataset revealed significantly higher expression of IFN-stimulated genes (ISG) signature in MSI-H GC compared to other subtypes of GC (Fig. 2E). Additionally, the expression of STING downstream T-cell attracting chemokines, including *CXCL9/10/11* and *CCL5*, was significantly elevated in MSI-H GC compared to other subtypes of GC with EBV (−) (Fig. 2F). These results suggest that STING signaling might be activated in dMMR GC, and STING downstream T-cell attracting chemokines, such as CXCL9/10/11 and CCL5, might play a role in CD8^+^ T-cell infiltration.

### Difference of the tumor cell-intrinsic expression of cGAS–STING among pMMR/EBV (−), dMMR, and EBV (+) GCs

**Fig. 3:**
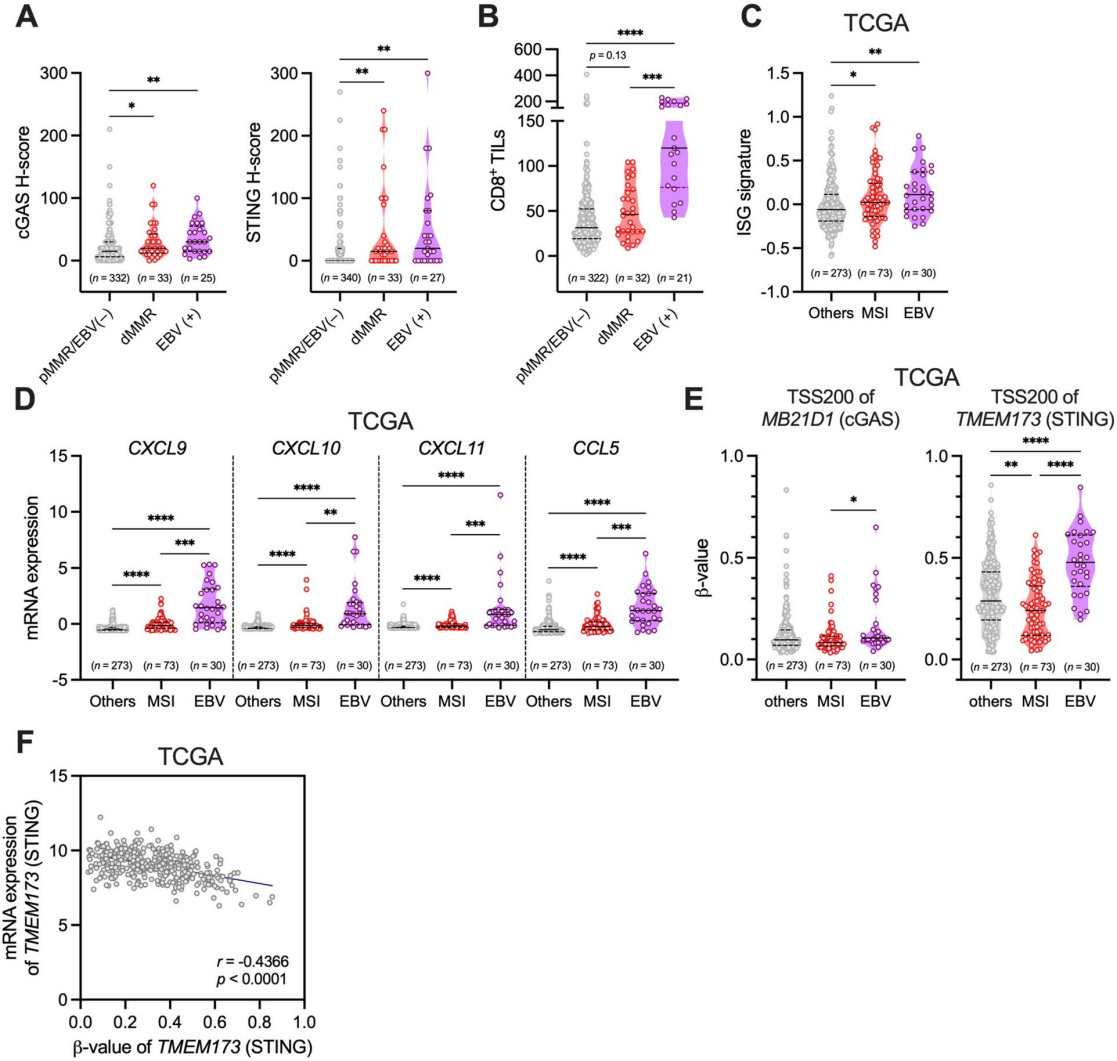
Difference in the tumor cell-intrinsic expression of cGAS–STING among pMMR/EBV (−), dMMR, and EBV (+) GCs. (**A**) Comparison of H-scores of cGAS–STING among pMMR/EBV (−), dMMR, and EBV (+) GCs. Solid lines represent medians, while dotted lines represent quantiles. (**B**) Comparison of the number of CD8^+^ TILs among pMMR/EBV (−), dMMR, and EBV (+) GCs. (**C**) Comparison of the expression of ISG signature among MSI, EBV, and other subtypes of GC with EBV (−) (TCGA cohort). (**D**) Comparison of the expression of *CXCL9/10/11* and *CCL5* among MSI, EBV, and other subtypes of GC with EBV (−) (TCGA cohort). (**E**) Comparison of β-values of *MB21D1* (cGAS) and *TMEM173* (STING) among MSI, EBV, and other subtypes of GC with EBV (−) (TCGA cohort). (**F**) Correlation between mRNA expression of *TMEM173* (STING) (log_2_ signal intensity) and the DNA methylation level (β-values) of the promoter region of *TMEM173* (STING) in GC, including MSI GC, EBV GC, and other subtypes of GC with EBV (−) (TCGA cohort). *r*, correlation coefficient. Statistical significance was determined by the Kruskal–Wallis test with Dunn’s multiple comparisons test (**A**–**E**), and the correlation coefficient (*r*) was determined using the Spearman correlation test (**F**). *p < 0.05, **p < 0.01, ***p < 0.001, ****p < 0.0001.

It is well known that EBV (+) GC is a tumor exhibiting immune cell activation and high expression of PD-L1 in the TME^4,19^. When comparing the clinicopathological characteristics among pMMR/EBV (−), dMMR, and EBV (+) GCs, tumor location differed significantly among these three groups (Table 1). Moreover, the frequency of PD-L1-positive cases was significantly higher in EBV (+) GC compared to the other two groups (Table 1). The tumor cell-intrinsic expression of cGAS–STING was significantly higher in dMMR and EBV (+) GCs compared to pMMR/EBV (−) GC (Fig. 3A). The number of CD8^+^ TILs was also higher in dMMR GC (*p* = 0.13, not significant) and EBV (+) GC (*p* < 0.0001) compared to pMMR/EBV (−) GC, with the significant highest count observed in EBV (+) GC (Fig. 3B). Consistent with this results in our own cohort, the expression of ISG signature and STING downstream T-cell attracting chemokines, including *CXCL9/10/11* and *CCL5*, was significantly higher in MSI-H and EBV (+) GCs compared to other subtypes of GC with EBV (−) in the TCGA cohort (Fig. 3C, D). The DNA methylation of the promoter region of cGAS–STING might be involved in the down-regulation of these expressions in several cancers^20^. We found that the DNA methylation levels of the promoter region of *MB21D1* (cGAS) were comparable between other subtypes of GC and dMMR or EBV (+) GCs, whereas the DNA methylation level of the promoter region of *TMEM173* (STING) was significantly lower in dMMR GC compared to other subtypes of GC with EBV (−) and EBV (+) GC in the TCGA cohort (Fig. 3E). The DNA methylation level of the promoter region of *TMEM173* (STING) was significantly and inversely correlated with mRNA expression of *TMEM173* (STING) in GC (Fig. 3F), suggesting that the lower frequency of the DNA methylation might contribute to the higher expression of tumor cell-intrinsic STING in dMMR GC, but not in EBV (+) GC.

Taken together, dMMR GC, as well as EBV (+) GC, exhibit high expression of tumor cell-intrinsic cGAS–STING, which might contribute to the immune-active TME through increased infiltration of CD8^+^ T cells. Particularly, the low level of the DNA methylation of STING might be involved in its high expression in dMMR GC.

## Discussion

In this study, for the first time, we revealed that the tumor cell-intrinsic cGAS–STING is highly expressed in dMMR GC compared to pMMR/EBV (−) GC, and the high expression of tumor cell-intrinsic STING is significantly associated with the high infiltration of CD8^+^ T cells in dMMR GC in our own cohort. Moreover, based on the analysis of the TCGA cohort, the expression levels of IFN-stimulated genes and STING downstream T-cell attracting chemokines, including *CXCL9/10/11* and *CCL5*, were significantly higher in MSI-H GC compared to other subtypes of GC with EBV (−), suggesting that the activation of STING signaling and its downstream T-cell attracting chemokines might contribute to the high infiltration of CD8^+^ T cells in dMMR/MSI-H GC.

It is widely accepted that dMMR/MSI-H tumors exhibit a favorable response to ICI due to high tumor mutation burden and neoantigen load, resulting in the activation of immune cells, including CD8^+^ T cells, in the TME. Our previous study suggested that dMMR/MSI-H colorectal cancer (CRC) exhibited higher expression of tumor cell-intrinsic cGAS–STING, concomitant with higher infiltration of CD8^+^ TILs, compared to pMMR/MSS CRC^21^. Additionally, in our present study, the same trend was observed in dMMR/MSI-H GC compared to pMMR/MSS/EBV (−) GC. Therefore, the tumor cell-intrinsic cGAS–STING could be a crucial component for the activation of immune cells in the TME of dMMR/MSI-H gastrointestinal adenocarcinomas. Furthermore, our current findings suggest that the expression of tumor cell-intrinsic cGAS–STING, along with MMR/MSI status, could be a novel biomarker predicting the efficacy of immunotherapy using ICIs in GC.

In contrast to dMMR/MSI-H GC, the expressions of tumor cell-intrinsic cGAS–STING, IFN-stimulated genes, and STING downstream T-cell attracting chemokines were significantly lower in pMMR/MSS/EBV (−) GC (Figs. 1 and 2), suggesting that the activation of the tumor cell-intrinsic cGAS–STING is maintained at a low level in pMMR/MSS/EBV (−) GC. Targeting the cGAS–STING pathway has been considered a novel therapeutic strategy to improve clinical efficacy for cancer immunotherapy^22^, and several clinical trials utilizing STING agonists, such as 5,6-dimethylxanthenone-4-acetic acid, ADU-S100, and MK-1454, in solid tumors have been completed and/or ongoing^23^. A preclinical study has demonstrated that intratumoral injection of ADU-S100 induced CD8^+^ T-cell-mediated anti-tumor immunity, and the combination of ADU-S100 with ICIs induced synergistic effects in the expansion of CD8^+^ T cells and durable eradication of tumors in in vivo mouse tumor models^24–26^. Furthermore, a combination therapy using MK-1454 and anti-PD-1 antibody showed a 24% response rate with reductions in the size of both target-injected and noninjected lesions (83% median) in advanced solid tumors^27^. Therefore, combination therapy utilizing STING agonists with ICIs might be applicable to enhance the efficacy of treatment in pMMR/MSS/EBV (−) GC.

A direct link between high STING expression in tumor cells and increased CD8^+^ T cell infiltration was not observed in this study. Vornholz et al. demonstrated that in mouse models, the growth of subcutaneously transplanted dMMR tumors was significantly slower than that of pMMR tumors. This slower growth was associated with upregulated expression of T cell-attracting chemokines, such as CXCL10, and higher frequencies of CD8^+^ T cells in the TME. Moreover, the deletion of STING in tumor cells markedly reversed the dMMR-mediated reduction in tumor growth, as well as the elevated levels of CXCL10 expression and increased recruitment of CD8^+^ T cells in the TME ^28^. Furthermore, blocking type I IFN signaling or the CXCL10–CXCR3 axis, which mediates the recruitment of CD8^+^ T cells to the TME, led to more aggressive growth of dMMR tumors^28^. These findings suggest that tumor cell-intrinsic STING signaling contributes to creating an immune-active, cytotoxic T cell-enriched TME through mechanisms involving the type I IFN response and the CXCL10–CXCR3 axis in dMMR tumors. Indeed, our TCGA analysis showed higher expression of ISG signature and STING downstream chemokines, such as *CXCL10*, in MSI-H GC compared to other subtypes of GC with EBV (−) (Fig. 2E, F). Therefore, high expression of tumor cell-intrinsic STING might induce higher infiltration of CD8^+^ T cells through similar mechanisms in dMMR/MSI-H GC.

The regulatory mechanism behind the up-regulation of the tumor cell-intrinsic cGAS–STING expression in dMMR GC, as well as EBV (+) GC, remains unknown. Concerning STING expression, the DNA methylation level of STING was significantly lower in MSI-H GC compared to other subtypes of GC, including EBV (+) GC, suggesting that the low frequency of the DNA methylation of *TMEM173* (STING) might be one of the regulatory mechanisms contributing to the high expression of STING in dMMR/MSI-H GC. On the other hand, the DNA methylation level of STING is significantly higher in EBV (+) GC compared to other subtypes of GC, including MSI-H GC, implying that other regulatory mechanisms might be involved in the up-regulation of STING in EBV (+) GC. In EBV (+) GC, the EBV genome could directly activate the cGAS–STING pathway to induce a type I IFN response in GC cells^29,30^. It has been reported that STING is an IFN-stimulated gene and STING induction might be crucial for the positive feedback regulation of type I IFN^31^. Therefore, the high expression of STING might be regulated by the positive feedback loop of the type I IFN response triggered by the EBV genome in GC cells. Additionally, a previous study reported that the evaluation of the immune microenvironment score (IMS) of 1,422 GC samples, based on 51 immune cell signatures, revealed that GCs with a high IMS, including MSI-H and EBV (+) GCs, had not only abundant but also activated innate and adaptive immune cells compared to GCs with a low IMS, including CIN and GS GCs. These immune cells included activated CD4^+^ and CD8^+^ T cells, activated natural killer cells, and activated dendritic cells (DCs)^32^. Among these immune cells, tumor-infiltrating DCs are major players in both the production of and response to type I IFN in the TME^14,33^. Interestingly, Schadt et al. demonstrated that cancer cell-derived cGAMP can be transferred to DCs, further activating STING signaling and producing type I IFN in DCs in the TME^34^. Therefore, the high amount of type I IFN produced by activated DCs might also contribute to the high expression of STING in tumor cells in MSI-H and EBV (+) GCs. However, further investigations regarding the regulation of cGAS–STING expression in tumor cells, particularly in dMMR GC, are needed.

In conclusion, we have demonstrated that the tumor cell-intrinsic cGAS–STING pathway is associated with increased infiltration of CD8^+^ T cells in dMMR/MSI-H GC. Our current findings suggest the potential of tumor cell-intrinsic cGAS–STING as a novel biomarker for predicting the efficacy of immunotherapy using ICIs in GC and might provide new insights for treating patients with GC by targeting the tumor cell-intrinsic cGAS–STING pathway.

## Methods

### Patient samples

We recruited patients with GC who underwent surgical resection at Fukushima Medical University (FMU) Hospital between 2003 and 2019 from a total of consecutive GC cases (*n* = 401). The total number of cases was divided into three groups: pMMR/EBV (−) GC (*n* = 341), dMMR GC (*n* = 33), and EBV (+) GC (*n* = 27). Clinicopathological information was retrospectively collected by reviewing medical records. This study was approved by the Institutional Ethics Committee of FMU (Reference No. 2329), and all procedures were conducted following the principles outlined in the Helsinki Declaration. Written informed consent to participate in the study was obtained from all participants.

### IHC analysis

Paraffin-embedded 4-μm sections of GC tissue were deparaffinized in xylene and rehydrated in ethanol. Endogenous peroxidases were blocked using 0.3% hydrogen peroxide in methanol. Antigens were retrieved by autoclaving with Target Retrieval Solution at pH 6.0 or pH 9.0 (Dako/Agilent Technologies, Santa Clara, CA). Following PBS washing, the sections were incubated overnight at 4 °C with the following primary antibodies: anti-cGAS monoclonal antibody (#79978; dilution 1:300), anti-STING monoclonal antibody (#13647; dilution 1:200) (Cell Signaling Technology, Danvers, MA), and anti-CD8 monoclonal antibody (M7103; dilution 1:400) (Dako/Agilent Technologies). Subsequently, the sections were incubated with HRP-conjugated anti-mouse or anti-rabbit secondary antibodies (K4003 or K4001, Dako/Agilent Technologies). Peroxidase activity was visualized using diaminobenzidine, and nuclei were counterstained with Mayer Hematoxylin Solution.

### Assessment of IHC

A total of 391, 400, and 376 samples were used to assess the expression of cGAS, STING, and CD8, respectively. The expression of cGAS and STING in tumor cells was evaluated using the IHC score (H-score; 0–300), calculated by multiplying the intensity score and extent score. The intensity score was graded based on staining in the cytoplasm as follows: 0 (none), 1 + (weak), 2 + (moderate), or 3 + (strong), while the extent score represented the percentage of stained cytoplasm (0–100%)^35^. To assess the presence of cGAS–STING in tumor cells, we reassessed the extent score using the following criteria: 0 for no staining at all, 1 for < 10%, 2 for 10–50%, and 3 for > 50% of tumor cells stained. The final score, ranging from 0 to 9, was determined by multiplying the reevaluated extent score (0–3) and intensity score (0–3). Cases with a final score of 3 or higher were classified as cGAS high (cGAS^high^) and STING high (STING^high^)^35^. For assessing CD8^+^ tumor-infiltrating lymphocytes (TILs), the tumor core of GC tissues was reviewed in four independent areas, as previously described^35^. IHC analyses of MMR, human epidermal growth factor receptor 2 (HER2), and PD-L1 combined positive score (CPS), and an in situ hybridization analysis of the integrated EBV genome were performed as previously reported^35–37^. Evaluation of IHC was conducted by three observers (R.K., S.N., and S.F.) who were blinded to all clinical and pathological information. Samples with discrepancies between the observers were discussed and reevaluated jointly until consensus was reached.

### Data analysis of The *Cancer* Genome Atlas (TCGA) database

We acquired publicly accessible datasets containing patients' clinicopathological information, mRNA expression of *CXCL9/10/11*, and *CCL5*, and ISG (the human gene set “GOBP_RESPONSE_TO_TYPE_I_INTERFERON” from MSigDB], as well as DNA methylation of *MB21D1* (cGAS) and *TMEM173* (STING) for stomach adenocarcinoma (TCGA STAD; *n* = 440) from cBioPortal (https://www.cbioportal.org)^38^. We compared mRNA expression levels of *CXCL9/10/11* and *CCL5*, the average of STING signaling genes, and methylation levels of *MB21D1* (cGAS) and *TMEM173* (STING) (β-values) among EBV (+) GC (EBV; *n* = 30), MSI-H GC (MSI; *n* = 73), and other subtypes of GC with EBV (−), including GS (*n* = 50) and CIN (*n* = 223). Cases with not available for the information of GC subtype (*n* = 57) and *POLE* mutation (*n* = 7) were excluded from this analysis. Z-scores were used for all gene expression analyses, unless otherwise indicated.

### Statistical analysis

The data are presented as means ± SD. Statistical analyses were performed using GraphPad Prism 9 version 9.5.1 (GraphPad Software, San Diego, CA). Two-group comparisons of means were conducted using the Mann–Whitney U test. For comparisons involving multiple groups, we utilized the Kruskal–Wallis test with Dunn’s multiple comparisons test. Proportions of groups in categorical variables were compared using Fisher’s exact test or the Chi-square test. The correlation was analyzed using the Spearman rank-correlation coefficient. A *p*-value less than 0.05 was considered statistically significant.

## Data Availability

The datasets generated and/or analyzed during this study are available from the corresponding author on reasonable request.
